# Relationship between the duration of ischemia and final infarct size in STEMI patients treated with abciximab and primary PCI

**DOI:** 10.1186/1532-429X-14-S1-P21

**Published:** 2012-02-01

**Authors:** Tim Tödt, Eva Maret, Joakim Alfredsson, Magnus Janzon, Jan E Engvall, Eva E Swahn

**Affiliations:** 1 Department of Medical and Health Sciences, Division of Cardiology, Linköping University, Linköping, Sweden; 2 Center for Medical Image Science and Visualization, Linköping University, Linköping, Sweden; 3 Department of Clinical Physiology, Ryhov County Hospital, Jönköping, Sweden; 4 Department of Clinical Physiology UHL, Linköping University, Linköping, Sweden

## Summary

We studied 89 patients with first time myocardial infarction and treated with primary PCI. There was only a weak correlation between ischemia duration and final myocardial scar size measured with ceMRI six weeks later. Other factors like anterior infarction, a patent artery pre-PCI and effects of reperfusion injury may have had greater influence on infarct size than time-to-treatment per se.

## Background

Studies on the impact of time to treatment in primary percutaneous coronary intervention (PCI) on myocardial infarct size have yielded conflicting results. We investigated the relation between the size of myocardial injury and time from symptom onset to demonstration of an open infarct related artery (IRA) in ST-elevation myocardial infarction (STEMI) treated with primary PCI.

## Methods

Between February 2006 and September 2007 we studied 89 STEMI patients treated with primary PCI and abciximab. Contrast enhanced magnetic resonance imaging (ceMRI) was performed 4-8 weeks after the acute event. Spearman correlation was computed for the potential relationship between ischemic time and myocardial injury. Multiple linear regression was used to determine independent correlates of infarct size.

## Results

An occluded artery (Thrombolysis In Myocardial Infarction, TIMI flow 0-1 at initial angiogram) was seen in 56 patients (63%). Abciximab was given to all but one patient and 79 % were pre-treated with abciximab before arriving at the cath-lab. The median time to a patent artery was 193 minutes. There was a weak correlation between time from symptom onset to an open culprit artery and infarct size, r=0.21, p=0.05. Patients with a patent artery within two hours of symptom onset appeared to have smaller infarct size in absolute and relative terms than those with longer ischemic times although this did not reach statistical significance. In multiple regression analyses LAD as the IRA, smoking and an occluded vessel at the first angiogram, but not ischemic time, correlated with infarct size.

## Conclusions

In patients with STEMI treated with primary PCI we found only a weak correlation between ischemic time and infarct size. Since mean duration of ischemia was more than three hours, other factors like anterior infarction, a patent artery pre-PCI and effects of reperfusion injury may have had greater influence on infarct size than time-to-treatment per se.

## Funding

This work was supported by the Medical Research Council of Southeast Sweden, the medical faculty of Linköping University, the Swedish Heart-Lung foundation, the Swedish Research Council, Futurum - the Academy for health Care Jönköping County Council and the Center of Medical Image Science and Visualization at Linköping University Hospital.

**Figure 1:**
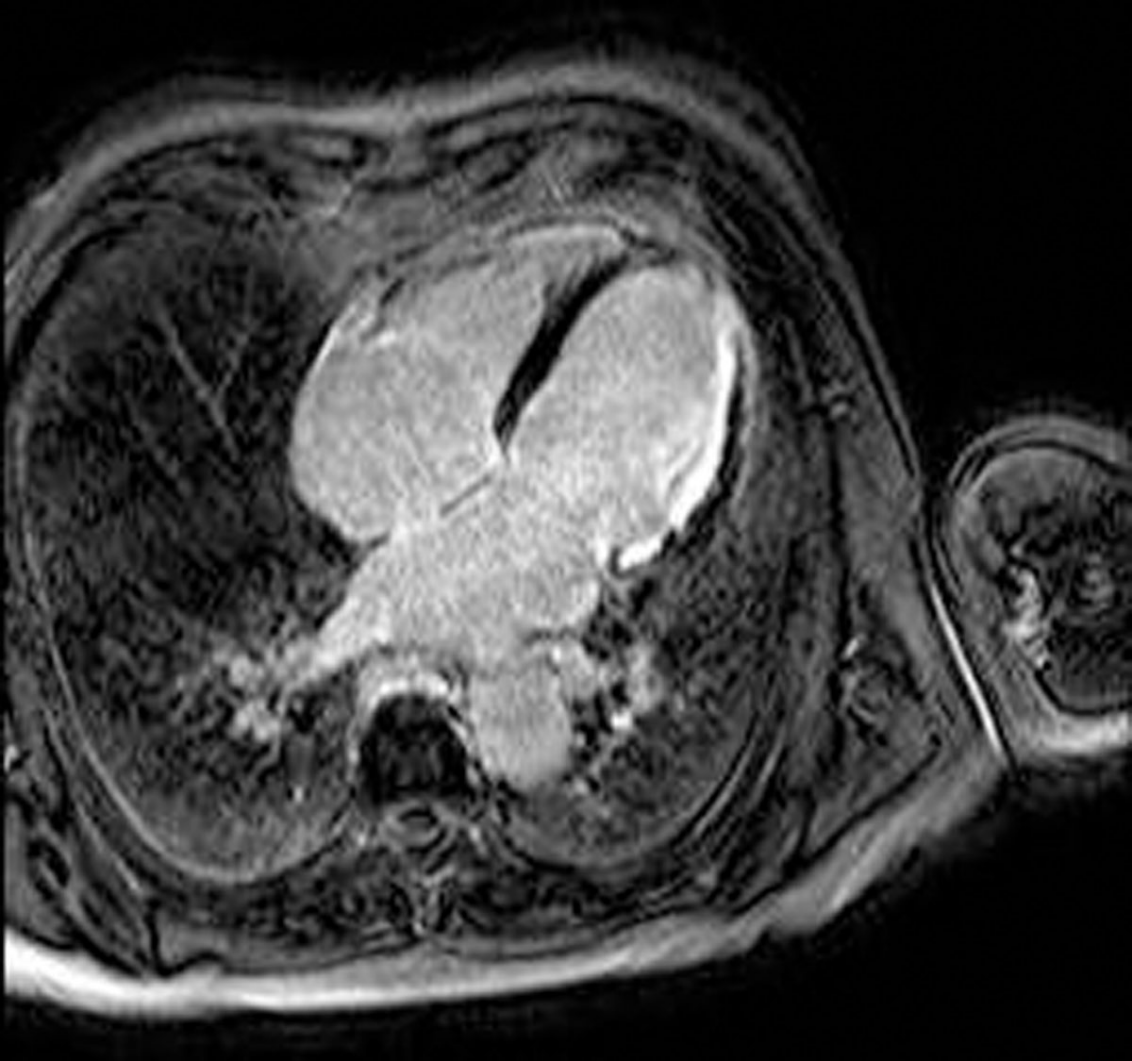
Subendocardial extent of infarct scar in the lateral wall of the left ventricle six weeks post PCI.

**Table 1:** Multiple linear regression. Predictors of infarct size (Scar ml).

Variable	B (95% CI)	P value
Age	0.27 (-0.39-0.59)	.085
Occluded artery at first angiogram	10.9 (5.2-16.5)	.0005
LAD as culprit artery	10.2 (4.8-15.6)	.0005
Symptom to patent artery (minutes)	0.18 (-0.54-0.89)	.62
Active smoker	7.9 (2.1-13.7)	.008
Male sex	4.9 (-2.7-12.4)	.20
Hypertension	3.9 (-2.2-10.2)	.20
Diabetes	0.26 (-11.5-12.0)	.96

B, regression coefficient. CI, confidence interval. LAD, left anterior descending artery.

